# Long-Term Effects of Transtheoretical Model-Based Lifestyle Intervention on Self-efficacy and Self-management in Patients with Type 2 Diabetes — Randomised Controlled Trial

**DOI:** 10.1007/s12529-024-10323-0

**Published:** 2024-09-30

**Authors:** Annalena Dunkel, Katja von Storch, Martin Hochheim, Susanne Zank, Maria Cristina Polidori, Christiane Woopen

**Affiliations:** 1 NRW Graduate School GROW - Gerontological Research on Well-Being, Faculty of Medicine and Faculty of Human Sciences, University of Cologne, Albertus-Magnus-Platz, 50923 Cologne, Germany; 2 Generali Health Solutions GmbH, Cologne, Germany; 3 Rehabilitative Gerontology, Faculty of Human Sciences, University of Cologne, Cologne, Germany; 4 Department II of Internal Medicine and Center for Molecular Medicine Cologne, Ageing Clinical Research, University of Cologne, Faculty of Medicine and University Hospital Cologne, Cologne, Germany; 5 Cologne Excellence Cluster on Cellular Stress- Responses in Aging- Associated Diseases (CECAD), University of Cologne, Faculty of Medicine and University Hospital Cologne, Cologne, Germany; 6 Center for Life Ethics, University of Bonn, Bonn, Germany

**Keywords:** Lifestyle modification, Type 2 diabetes, Telemedicine, Self-management, Hb_A1c_

## Abstract

**Background:**

Self-efficacy and self-management are fundamental factors for successful treatment of type 2 diabetes, but long-term studies are rare. The aim of the present study is to investigate whether the effects achieved in the context of a lifestyle intervention based on the transtheoretical model can be maintained by the patients in the long term.

**Method:**

A two-arm randomised controlled trial examined whether long-term effects of self-efficacy, self-management, and Hb_A1c_ can be achieved by a lifestyle intervention of 12 months and persisted beyond the intervention. During the intervention, the intervention group (*n* = 86, mean age 59.7 years) was supported by a telephone coach and telemedical devices, while the control group (*n* = 65, mean age 58.8 years) received regular care. In the year after intervention, both groups received standard care.

**Results:**

The intervention group achieved significantly better self-management after 12 and 24 months (12M, 1.11 (0.81; 1.41) *p* < .000; 24M, 0.52 (0.19; 0.85) *p* = .002) as well as self-efficacy (12M, 1.18 (0.83; 1.52) *p* < .000; 24M, 0.76 (0.39; 1.13) *p* < .000) and Hb_A1c_ than the control group.

**Conclusion:**

TTM-based lifestyle interventions show a long-term effect beyond the duration of the intervention in most areas, and behavioural changes can be sustained by patients.

## Introduction

Diabetes mellitus (DM) is one of the four most common chronic diseases worldwide. It currently affects around 61.4 million people in Europe, and the prognosis continues to rise [1]. A major problem is the numerous comorbidities and secondary diseases that are associated with diabetes, which are directly or indirectly responsible for about 12.2% of all deaths of people aged 20 to 79 worldwide [1]. In addition to the burden on those affected, DM also places an immense burden on global healthcare systems; therefore, prevention and healthcare are becoming increasingly important [2, 3].

Numerous studies have shown that diabetes self-management, which includes adjustments to diet, exercise, medication, and self-monitoring, among other factors, is a fundamental factor in the successful treatment of type 2 diabetes (T2DM), which accounts for 90% of all DM cases. Self-management is defined in this study in accordance with the “Deutsche Diabetes Gesellschaft” (DDG; German Diabetes Association) as the competence to independently organise and manage life with the chronic disease diabetes according to one’s own goals and values and includes the ability to set goals and successfully implement them strategically [4]. In addition to successful treatment, which may even result in remission of T2DM, self-management and behavioural interventions also play a role in preventing iatrogenic reactions and adverse side effects, as well as positively influencing the quality of life and general health [5–14]. However, studies have also shown that diabetes self-management by those affected is very often not optimal, and it is also known that many people have great difficulty adopting new behaviours in their daily lives over the long term [15–17]. The non-adherence rate of lifestyle interventions is 40 to 80% and the dropout rate is between 30 and 60% [17–19]. This is a rather disturbing situation, as long-term improvements are usually due to long-term maintenance.

It is believed that self-efficacy leads to better self-management and, especially, long-term maintenance of the behavioural changes as well as lower Hb_A1c_ and better quality of life [20–22]. Interventions that work only with knowledge transfer have not achieved satisfactory effects in securing long-term maintenance of the learned behaviour [23]. The construct of self-efficacy originally comes from social cognitive theory (SCT) and, in contrast to self-management, is not defined by real abilities but by the individual’s own judgement of their own abilities and therefore always relates to a specific situation. Due to this situational dependency, self-efficacy is considered to be relatively easy to influence when it is about changing behaviour [24, 25]. Now, self-efficacy is included in various theoretical constructs, such as the transtheoretical model (TTM), an integrative model of behaviour change that can be used to predict a patient’s readiness to implement a proposed behaviour change [26, 27]. Motivational interviewing is a method that has already shown good results in interventions based on the transtheoretical model with type 2 diabetics [28, 29]. Self-control and flexible treatment recommendations based on individual preferences as well as social support and long-term professional contacts, on the other hand, have so far been shown to be relevant factors leading to improved self-management [15, 30–33]. It is also known that theory-based interventions usually lead to more satisfactory outcomes for patients with type 2 diabetes compared with non-theory-based interventions [23, 34]. However, the results regarding self-efficacy based on educational interventions for T2DM refer to short interventions and follow-up periods. The most common studies on self-efficacy have an average length of fewer than 3 months and follow-up periods between 3 and 6 months [23]. Studies over a longer period are comparatively rare. As it is assumed that self-efficacy has a positive influence on the long-term maintenance of behavioural changes, more high-quality studies over long periods are needed to investigate this assumption [35, 36].

We aim to address this research gap by using real-world evidence from a long-term intervention with an additional follow-up of a telemedicine-assisted lifestyle diabetes programme called initiative.diabetes compared to routine care in Germany. The intervention combines telemedically supported self-monitoring with flexible and individualised recommendations by a personal coach in long-term professional support over 1 year. Data are from a randomised controlled trial (RCT) with a total duration of 2 years, comprising 1 year in the initiative.diabetes programme and a further year without support, after which a follow-up survey was conducted. Thus, statements can be made about long-term effects after 12 and 24 months. Self-management, self-efficacy, and health-related quality of life were used as explicit parameters. The initiative.diabetes programme evaluated in this CT is based on Prochaska’s TTM of behaviour change and incorporates many of the known theoretical parameters mentioned above.

### Theoretical Framework

Prochaska’s TTM aims to establish long-term behaviour change because of its dynamic nature [29]. The TTM is an intentional stage model for behaviour change and is one of the most widely used models in health behaviour. The model was developed at the end of the 1970s at the University of Rhode Island and integrates various (psycho-)therapeutic principles and strategies from different models, which is why it is also called “transtheoretical”. The original TTM comprises five successive stages of change in behaviour (SOC). In later models, a sixth stage is increasingly added [26, 37]. The stages are referred to as precontemplation, contemplation, preparation, action, maintenance, and termination, with the last three phases playing a predominant role in the present study. Precontemplation describes the stage in which people have no intention of changing their behaviour, contemplation refers to the consideration of a future change in behaviour, preparation describes the preparation, action is the active change in behaviour, and maintenance is the continuation of the changed behaviour (for at least 6 months). The sixth stage, termination, which is often added, describes the absolute confidence that the behaviour will be maintained in the long term and that the person will not relapse.

In addition, the original model includes the concept of self-efficacy, which shows a direct influence on health-promoting behaviour. In the context of TTM, self-efficacy is understood as “people’s situation-specific confidence that they can cope with risky situations without falling back into their unhealthy or risky habit” [26] and originates from a concept by Bandura [38].

Also, the technique of motivational interviewing, which is based on TTM, is used during coaching. Motivational interviewing is an evidence-based coaching model for health professionals to help patients achieve and implement set goals [29, 39]. Studies on the use of motivational interviewing in patients with T2DM showed good results and positive effects on self-efficacy so far [28, 40]. Furthermore, studies have shown that most patients who participate in TTM-based interventions reach the maintenance phase and achieve good outcomes in self-management behaviours, such as adherence to diet or increasing physical activity, especially patients with type 2 diabetes. No positive effects have yet been observed in medication adherence [41].

## Methods

### Study Design

The study was approved by the Ethics Committee of the Medical Faculty of the University of Cologne (Project ID, 17-021) and is registered with the German Clinical Trials Registry (DRKS00013737). It ran for a total period of 3.5 years (March 2017 to July 2020) as a two-arm randomised controlled intervention study in cooperation with a private German health insurance company (formerly, Central Krankenversicherung AG; since 2020, Generali Deutschland Krankenversicherung AG). All the participants consented to data collection and data processing for study purposes. Participants in the control group continued to receive standard care and were not an active or waiting control group. Standard care is the standard treatment provided by the general practitioner (GP) and includes the antidiabetic treatment algorithm based on the clinical practice guidelines of the DDG and the German Society of Internal Medicine (Deutsche Gesellschaft für Innere Medizin; DGIM). These are based on the National Disease Management Guideline (Nationale Versorgungsrichtlinien; NVL) “Type 2 Diabetes” [42–44]. Only participation in another structured programme outside of standard care was excluded by using a questionnaire. Participants in the intervention group participated in the initiative.diabetes programme for 1 year (see the following section). In the follow-up year, both groups received standard care.

### Study Population

**Fig. 1:**
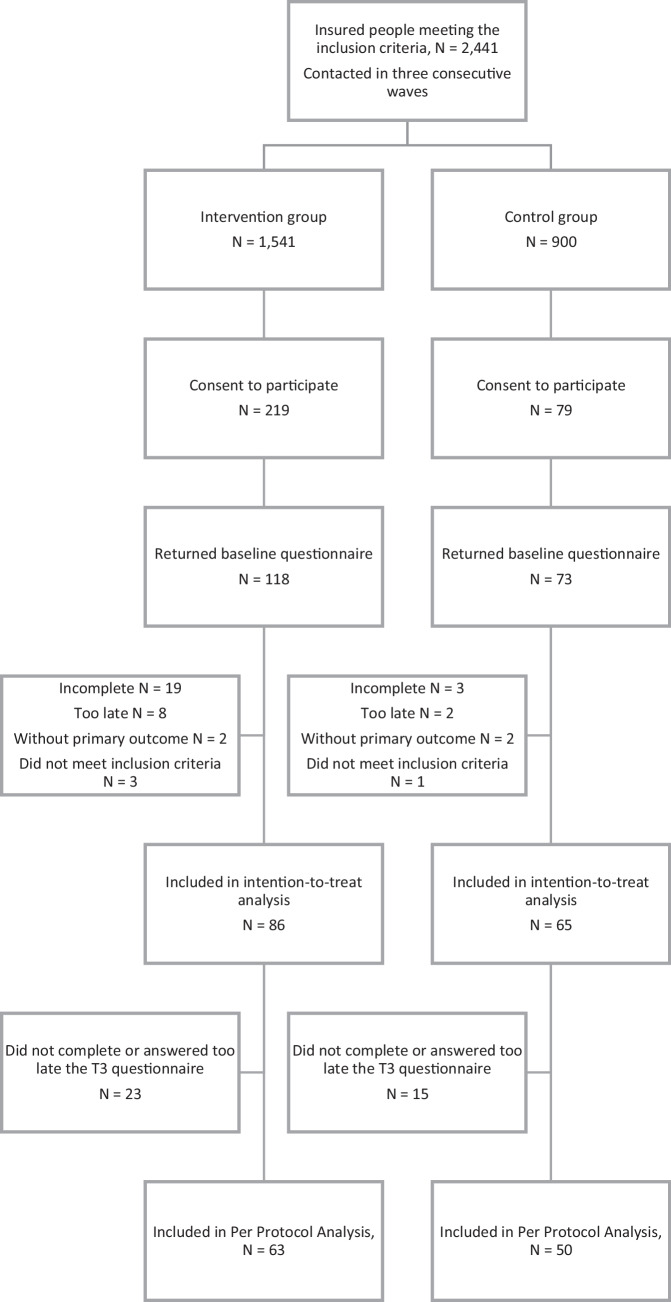
Consort flow chart

Recruitment was conducted throughout Germany from March to May 2017 and in cooperation with a private health insurance company as previously described [45]. All insured persons of Central Krankenversicherung AG were screened for inclusion and exclusion criteria. Persons aged between 40 and 67 years, with an official T2DM diagnosis (ICD-10 code E11), who were not pregnant or undergoing cancer treatment, did not suffer from any other life-threatening disease as well as from cognitive or mobility impairments, and did not require long-term care were included (*N* = 2.441 people who met all the criteria). Patients were randomised to the control group (CG) or intervention group (IG) in a proportion of approx. 1.5:1 (IG:CG). The randomisation factor was chosen as the group allocation took place prior to study start and under the assumption of a higher participation rate among controls. After allocation to CG or IG using a Zelen Design [46], all potential participants were contacted with a request to participate in the study arm to which they were assigned not disclosing the “pre” randomisation step. Of these, 298 individuals agreed to participate. According to the informed consenting process, participants agreed to the recording and use of their data for research purposes. Informed consent was obtained from the IG for participation in the *initiative.diabetes* health programme and for a survey and evaluation of the effects at multiple time points, and from the CG for participation in a study on the long-term improvement of standard care for people with type 2 diabetes. IG and CG members completed identical online questionnaires at home at baseline and at each measurement time point thereafter, with the intervention group additionally receiving a registration code for participation in the *initiative.diabetes* programme at baseline and completing additional programme assessments at each measurement time point. A total of 151 individuals answered the baseline questionnaire and could be included in the study sample (Fig. 1).

### The initiative.diabetes Programme

The initiative.diabetes programme is offered to insurees of Generali Deutschland Krankenversicherung, a large private health insurance company in Germany, and provides participants with T2DM 1 year of telephone coaching by professional diabetes specialists trained in motivational interviewing, supported by various technical devices such as a blood glucose meter, a pedometer, and a tablet PC. It includes self-monitoring via the provided devices, as well as individualised recommendations for behaviour change by the coach. The coaching is carried out within the framework of several modules on different topics and is based on the stages of the transtheoretical model. The focus is always on adaptation to the needs of each individual patient. The values transmitted on a daily basis can also be viewed by the coach and used as a further basis for coaching.

### Data Collection and Measures

A total of five measurement time points were collected: one measurement at baseline (T0), two intermediate measurements after 3 months (T1) and 6 months (T2), one final measurement after 12 months (T3), and one follow-up survey after 24 months (T4). The outcome variables collected were Hb_A1c_, self-management, self-efficacy, and health-related quality of life. In the follow-up survey, the need for longer care was also asked. All values were determined by patient self-report online questionnaires except the Hb_A1c_ value at the time points T0 to T3. This was recorded by the telephone coaching provider.

Diabetes self-management was assessed through the diabetes self-management questionnaire (DSMQ) (*α* = 0.84). This includes 16 items to evaluate self-care activities related to glycemic control, such as glucose management, nutritional control, physical activity, and healthcare use. All items were rated on a 4-point scale, with answers given as 0, 1, 2, and 3 for evaluation. Two items were excluded if the patients did not need oral medication or insulin. All values were summarised, divided by the maximum possible value, and multiplied by 10 to get the sum scale as a global measure of self-care. The final value ranged between 0 and 10, with a higher value indicating better self-management [47].

Diabetes self-efficacy was assessed through the Stanford Education Research Center’s Self-Efficacy for Diabetes Scale (SED), an eight-item diabetes-related scale assessing how confident patients are in certain activities. All items are scored from 1 to 10, with a higher mean score indicating better self-efficacy. The internal consistency reliability of this scale is 0.828 [48].

Health-related quality of life was measured using the Short-Form 12 questionnaire (SF-12), a shortened form of the SF-36. SF-12 represents the SF-36 dimensions of general health perception, pain, vitality, and social functioning with one item each. The dimensions physical functioning, physical role functioning, emotional role functioning, and psychological well-being are represented by pairs of items [49]. Responses were coded as instructed in the user’s manual to obtain the Physical and Mental Component Score. Both scores range from 0 to 100, where 0 represents the poorest health status [50, 51]. The SF-12 has already been validated in many studies and is a well-established scale. In a process of normalisation with a population-representative sample for Germany, the psychometric quality could be demonstrated. The physical scale score has a corrected discriminatory power ≥ 0.67, and Cronbach’s *α* = 0.89. The corrected discriminatory power for the psychological scale score is ≥ 0.53; Cronbach’s *α* = 0.89 [49].

All participants were asked to have their respective Hb_A1c_ values determined by their general practitioner. The Hb_A1c_ level represents the average blood glucose concentration during the last 2 to 3 months and is one of the established gold standards for blood glucose monitoring [52–54].

In addition, the number of coaching contacts made in the year of the intervention was recorded. In the follow-up survey, participants were also asked whether they would have liked to receive longer support through professional diabetes coaches or the initiative.diabetes programme.

### Data Analysis

All analyses were performed using SPSS versions 26 and 28. The main analyses of the results were performed according to the intention-to-treat (ITT) principle. For this, all participants who answered the questionnaire at baseline and provided Hb_A1c_ values at T0 were included. Based on an MCAR assumption according to Little, missing data in this sample were not replaced by imputations, but indirectly accounted for by using linear models [55].

The mean and standard deviations of the study participants at baseline were calculated to represent baseline characteristics. The absence of group differences at baseline was determined using Fisher’s exact test (dichotomous variables) or the *X*^2^ test and *T*-test (continuous variables).

The primary outcomes of the main analysis were the change in self-efficacy and self-management levels after 12 and 24 months, both within groups and between IG and CG. Secondary outcomes were Hb_A1c_ and SF-12. Repeated-measures linear mixed models were used for each parameter separately, using random intercepts and fixed effects for the group (two levels), time (five levels), and the interaction between the two, as well as the baseline outcome value as covariates to correct potential baseline differences [56–59]. No other covariates (e.g. age and sex) led to model improvements, and so were not added to the final model. After checking for outliers, one extreme outlier was removed in the SF-12 model (deviation ≥ 3 SD). A covariance structure based on autoregressive first order (AR1) could be chosen for each model. The significance level was set at .05. Sensitivity analysis included models according to the ITT principle with the imputation of missing data for non-responders using the baseline observation carried forward principle and models performing a per-protocol analysis of the data [60]. We defined the per-protocol population as those who completed the full 12-month intervention, independently of missing intermediate values at 3 or 6 months or dropping follow-up at 24 months.

In a subgroup analysis of the intervention group, an independent *T*-test was used to compare whether individuals with higher self-efficacy at the end of the intervention had better self-management behaviour and Hb_A1c_ levels in the follow-up survey. For this purpose, the intervention group (missing values imputed by baseline carried forward method) was divided into two groups based on their median in a per-protocol analysis.

## Results

### Baseline

Included were *n* = 86 subjects (80.2% male, mean age 59.7 years) in the intervention group and *n* = 65 subjects (83.1% male, mean age 58.8 years) in the control group. Table 1 shows that the largest proportion of participants was male (*n* = 123, 81.45%). The primary endpoint Hb_A1c_ at baseline was 6.9% (SD 0.9) for the IG and 6.8% (SD 1.0) for the CG. No differences in demographics, medical history, antidiabetic medication, or health parameters at baseline were seen in either group (Table 1).

**Table 1:** Baseline characteristics

		*n*	Intervention group	*n*	Control group
Demographic variables					
Age at study start (years)	*M* (SD)	86	59.66 (6.24)	65	58.80 (7.33)
Gender (male)	*N* (%)	86	69 (80.2)	65	54 (83.1)
Higher education (university degree)	*N* (%)	86	15 (17.4)	65	18 (27.7)
Higher income (> €5000/month)	*N* (%)	59	11 (18.6)	53	15 (28.3)
Antidiabetic medications					
Oral medication only	*N* (%)	86	54 (62.8)	65	43 (66.2)
Insulin only	*N* (%)	86	0 (0)	65	2 (3.1)
Both (oral and insulin)	*N* (%)	86	11 (12.8)	65	13 (20.0)
Health history					
Duration of diabetes (years since diagnosis)	*M* (SD)	84	6.29 (3.83)	65	6.91 (3.86)
Age at diagnosis	*M* (SD)	84	53.12 (7.09)	65	51.46 (7.22)
Multimorbidity (>3 chronic diseases)	*N* (%)	86	84 (97.7)	65	64 (98.5)
Diabetes-related comorbidities	*M* (SD)	86	3.17 (1.96)	65	3.23 (1.99)
Health indicators					
Glycemic control (Hb_A1c_)^a^	*M* (SD)	86	6.94 (0.94)	65	6.83 (0.97)
BMI^b^ (kg/m^2^)	*M* (SD)	86	31.08 (7.05)	65	29.48 (4.53)
Physician contacts (in the year before study)	*M* (SD)	72	10.78 (6.86)	61	11.61 (6.43)
Physical activity					
> 3h of moderate physical activity per day	*N* (%)	86	37 (43.1)	65	21 (32.3)
Questionnaire baselines					
DSMQ^c^ (0–10)	*M* (SD)	83	7.05 (1.60)	64	6.60 (1.51)
SED^d^ (0–10)^*^	*M* (SD)	86	7.33 (1.61)	65	8.20 (1.15)
SF-12^e^; physical score (0–100)	*M* (SD)	86	48.59 (7.96)	65	48.92 (8.15)
SF-12; mental score (0–100)	*M* (SD)	86	52.58 (8.97)	65	50.70 (10.00)

Data are mean ± standard deviation (SD) values or numbers (%) as indicated; ^a^*Hb*_*A1c*_, glycated hemoglobin; ^b^*BMI*, body mass index; ^c^*DSMQ*, Diabetes Self-Management Questionnaire; ^d^*SED*, Self-Efficacy for Diabetes Scale; ^e^*SF-12*, Short-Form 12 Questionnaire

^*^p < 0.05 (using Fisher’s exact test for categorical variables and *X*^2^- and *T*-tests for continuous variables)

#### DSMQ

The intervention group also showed an increase of 1.3 points (on a scale of 1 to 10) in self-management behaviour and performed significantly better than the control group both after 12 months (1.11, 95% CI [0.81; 1.41], *p* < .000) and at follow-up after 24 months (−0.52, 95% CI [0.19; 0.85], *p* = .002).

#### SED

**Fig. 2:**
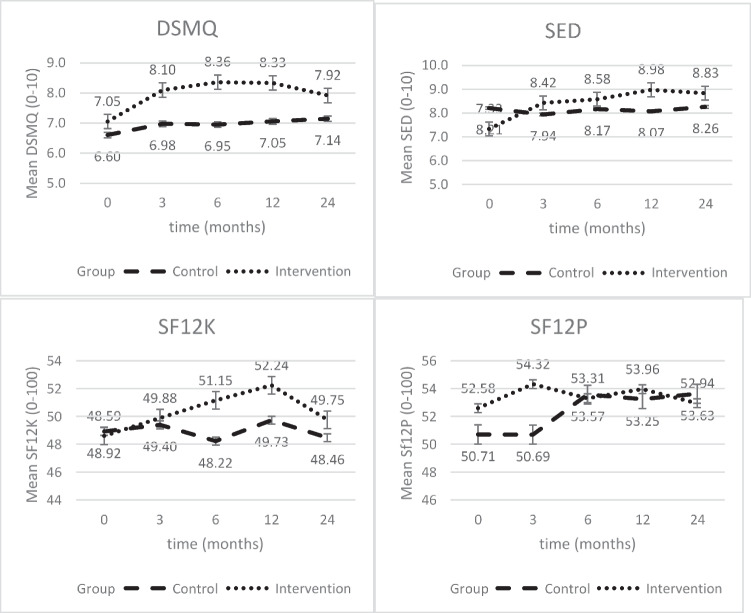
Development of DSMQ, SED, and SF-12 values at the whole time; data are the least-square means derived from the linear mixed model and adjusted for baseline values. Error bars indicate standard errors

In the self-efficacy score, the most significant increase was shown in the intervention group. In the beginning, this was still significantly below the value of the control group (the only baseline value with a significant difference). However, after both 12 months (1.18, 95% CI [0.83; 1.52], *p* < .000) and at follow-up after 24 months (0.76, 95% CI [0.39; 1.13], *p* < .000), the IG performed significantly better than the CG, especially in the first 3 months (Fig. 2), and was able to maintain a significant increase over the long term.

### Hb_A1c_

**Fig. 3:**
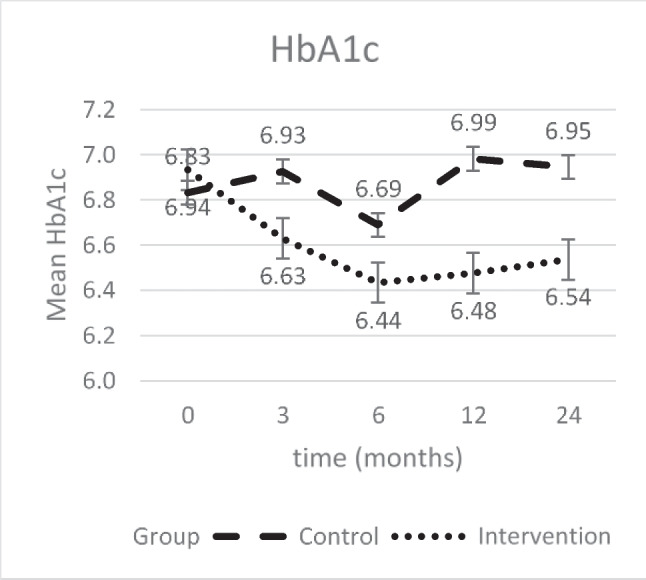
Development of Hb_A1c_ values over time; data are the least-square means derived from the linear mixed model and adjusted for baseline hemoglobin A1c. Error bars indicate standard errors

Significantly better Hb_A1c_ values were shown by the intervention group than by the control group both at the end of the intervention (−0.52, 95% CI [−0.73; −0.32], *p* < .000) and after 24 months (−0.38 (−0.61; −0.15), *p* = .001) (Table 2). Considering the development across the whole period, the strongest decrease can already be seen in the first 3 months of the intervention, while the lowest value is reached only after 6 months (Fig. 3).

**Table 2:** Mean changes in glycemic control (Hb_A1c_) self-management, self-efficacy, and health-related quality of life in the intervention and control groups across 12 months and 24 months

Outcome		Groups, mean change (95% CI)		Between group mean difference (95% CI)	*p* value of difference
		Intervention group (*n* = 86)	Control group (*n* = 65)		
Hb_A1c_^a^	12 M	−0.48 (−0.65; −0.31)**	0.05 (−0.15; 0.25)	−0.52 (−0.73; −0.32)	.000
	24 M	−0.39 (−0.58; −0.19)**	−0.01 (−0.22; 0.21)	−0.38 (−0.61; −0.15)	.001
DSMQ^b^	12 M	−1.42 (−1.66; −1.14)**	0.31 (0.04; 0.58)*	1.11 (0.81; 1.41)	.000
	24 M	−0.96 (−1.23; −0.69)**	0.44 (0.15; 0.74)*	0.52 (0.19; 0.85)	.002
SED^c^	12 M	1.36 (1.11; 1.62)**	0.19 (−0.10; 0.48)	1.18 (0.83; 1.52)	.000
	24 M	1.14 (0.86; 1.42)**	0.39 (0.08; 0.69)*	0.76 (0.39; 1.13)	.000
SF-12^d^ (PhSS^e^)	12 M	3.45 (2.04; 4.85)**	0.56 (−1.08; 2.21)	2.88 (1.14; 4.63)	.001
	24 M	1.16 (−0.46; 2.78)	−0.61 (−2.40; 1.15)	1.77 (−0.23; 3.78)	.083
SF-12 (PSS^f^)	12 M	1.58 (0.02; 3.14)*	2.00 (0.24; 3.76)*	−0.42 (−2.37; 1.54)	.675
	24 M	0.62 (−1.09; 2.33)	2.15 (0.30; 4.00)*	−0.53 (−3.69; 0.63)	.164

^a^*Hb*_*A1c*_, glycated hemoglobin; ^b^*DSMQ*, Diabetes Self-Management Questionnaire; ^c^*SED*, Self-Efficacy Scale for Diabetes; ^d^*SF-12*, Short-Form 12 Questionnaire; ^e^*PhSS*, Physical Sum Score; ^f^*PSS*, Psychological Sum Score

^*^p < .005; **p < .000

#### SF-12

The SF-12 results differed from the other results. The physical sum score (PhSS) showed a significant improvement in the IG compared to the CG. However, this positive change began later and was not yet detectable after 3 months, but only after 6 months (2.336; CI, 0.641; 4.032, *p* = .002). The physical cumulative score of the CG did not change significantly at any time. In contrast, the psychological sum score (PSS) in the IG showed no significant changes, while the CG showed significant positive effects.

### Subgroup Analysis: Effects of Self-efficacy on Long-Term Effects of the IG

Although both the median (9.0) and mean (8.57) self-efficacy scores at T3 were relatively high in the overall group, subgroup analysis of the IG showed that individuals who completed the intervention with a higher self-efficacy score still had significantly better blood glucose levels and self-management 1 year later than individuals with slightly lower self-efficacy. Hb_A1c_ was statistically 0.36 (95% CI [−0.69, −0.02] *t*(66) = 30.30, *p* = .002) lower and self-management was 1.02 (95% CI [0.40, 1.64] *t*(67) = −2.11, *p* = .038) better.

## Discussion

The present study investigated the change in self-management, self-efficacy, Hb_A1c_, and health-related quality of life after a 1-year lifestyle intervention and after a further year without professional support in a follow-up survey. Significant changes were found in glycemic control as well as in self-management and self-efficacy, whereas the health-related quality of life did not show statistically significant changes. The initiative.diabetes programme focused specifically on improving self-management and glycemic control, using theory-based self-efficacy techniques in particular. Improving health-related quality of life is not considered a key focus of the programme but rather an expected outcome.

The analysis of the demographic variables showed a relatively high level of education, as well as a disproportionate distribution male participants. However, the gender distribution is not likely to matter too much, as the general distribution of diabetes prevalence also shows slightly more males than females, although with a slightly smaller difference than in the present study [1]. The average age at diagnosis (approximately 52 years) of the sample is in line with the German average (53.2 years for men and 57.4 years for women), reflecting a normal expression. The average duration of diabetes is also of interest, as early detection and treatment are thought to lead to better therapeutic outcomes. The participants in the present study have now had diabetes for an average of 6.6 years.

Self-management in patients with type 2 diabetes is a popular research field, with numerous studies consistently demonstrating improvement in glycemic control (mostly measured by Hb_A1c_ level) in multiple RCTs, reviews, and meta-analyses. In the vast majority of studies, the Hb_A1c_ value is recorded as the primary outcome [9, 22, 61]. The few existing long-term studies also consistently show a decline in effects over time [11, 62]. This may be one of the reasons why the American Diabetes Association (ADA) recommends annual monitoring to determine whether further or renewed support is needed to improve self-management [63]. The exact cause of the decline is not clear from the studies. However, the results regarding parameters such as BMI, physical activity, self-efficacy, health-related quality of life, or other psychological parameters, which are recorded in different ways as secondary parameters in many studies, are not consistent, most probably due to the strong focus on glycemic control. Furthermore, many studies do not explicitly survey self-management at all, but present it by individual dimensions (diet, physical activity, foot care, etc.) or in terms of changes in glycemic control and imply it as an underlying mechanism [48, 64].

In the present RCT, an increase of 1.28 points (on a scale of 1 to 10) in self-management was achieved in the IG within 1 year. In contrast, the values in the control group showed only a slight, nonsignificant increase. After 24 months, there was a slight regression of the effect within the IG but still, a significantly better result than at baseline and compared to the control group. However, precise classification of the recorded scores is difficult in this area, as there are numerous different measurement instruments and study designs. For example, a recent Turkish study also used the DSMQ to investigate the effect of telephone counselling based on the information, motivation, behavioural literacy (IMB) model on Hb_A1c_ levels and self-management in patients with type 2 diabetes mellitus (T2DM) [65]. Significant improvements in the intervention group were also recorded (+2.33 points), which were significantly greater than in the present trial. However, the intervention duration was much shorter (3 months), and the baseline value of the study participants was considerably lower (3.88 ± 1.57), so there was a higher potential for increase [65].

The Hb_A1c_ value was also recorded in the present study. Hb_A1c_ showed a significant reduction both after 12 and after 24 months in the intervention group, while there were no significant changes in the control group. The recommendations for the Hb_A1c_ value are less than 7% according to the ADA [66]. Already at the beginning of the study, the mean Hb_A1c_ value was lower than the recommended value, but further improvements could be achieved. In general, however, this success is in line with the current literature [11, 67, 68].

The studies on the influence of self-efficacy on blood glucose control and better self-management, on the other hand, show no consistent findings. In part, no improvements were found; in part, there were clear direct effects; and in part, a mediating role is attributed to it [16, 22, 29, 69, 70]. Nevertheless, it is also repeatedly credited with a decisive influence on long-term outcomes and the longer-term maintenance of health behaviours [21].

With regard to self-efficacy in the present study, the course looks relatively similar to self-management, and significant effects can also be recorded here after 12 and 24 months. In the intervention group, an increase of 1.65 points (on a scale of 1 to 10) was achieved in the intervention year, which also shows a reduction of only 0.1 points 1 year later. This improvement was achieved even though relatively high self-efficacy scores (7–8 on a scale of 0 to 10) were already present at baseline. The decisive factor for this may have been the targeted use of the motivational interviewing technique, since this technique has already shown a clear, positive influence on self-efficacy [28, 29]. Similar to self-management, comparability is also difficult here due to numerous measurement instruments and designs. However, one study on the influence of TTM-based motivational interviewing on self-efficacy, health behaviour, and the Hb_A1c_ value, which is based on the same theory and using the same interviewing technique, showed a similarly high increase in self-efficacy when compared. Again, the duration of the study (6 months) differed, indicating no long-term values [29].

Based on the literature, it is also assumed that self-efficacy also has an impact on long-term maintenance [20, 22, 71, 72]. A subgroup analysis of the intervention group showed that, despite the overall very high scores, individuals with a higher self-efficacy score at the end of the intervention (score ≥ 9) had significantly better scores in self-management and Hb_A1c_ 1 year later in the follow-up survey than individuals with lower self-efficacy (score < 9). In a 2018 study, self-efficacy also emerged as the only significant determinant regarding adherence to required self-care behaviours. However, it should be noted that this was a cross-sectional study [21].

The evaluation of the health-related quality of life using SF-12 did not show as clear effects as the other parameters surveyed. In the IG, positive effects were found only in the area of physical health, but not in the area of mental health. This is confirmed by a recent study that, using the SF-36 (the long form of the SF-12), also found positive effects only in physical health–related quality of life, but not in mental health–related quality of life, after a 1-year lifestyle intervention [73]. In the CG, the opposite was the case. Moreover, these effects occurred later than the effects of the other outcomes (Fig. 2). One possible explanation for the missing changes in mental health in the IG is that diabetes usually does not cause constant pain and causes few to no limitations in the early stages [2]. Since the participants in this study were generally not insulin-dependent, and the initial values of Hb_A1c_ were already quite low at baseline, it can be assumed that the psychological distress was not very high and thus offered little room for improvement. This is supported by the fact that in another study of insulin-dependent patients with a longer duration of the disease, positive effects on mental health were found [74]. Both factors are associated with a worse health-related quality of life. Besides, the mean value of the German norm sample for the use of SF-12 in diabetics is 38.89 (±9.93) for the physical sum score and 50.10 (±9.81) for the psychological sum score [75]. The psychological sum score is almost identical to the baseline value of this study, which means that patients had already reached the norm at the beginning. A major change could not be expected. The change in the physical sum score is even more surprising since it was already well above the norm at baseline and yet, a significant improvement has occurred in the IG.

## Strengths and Limitations

The present study has some limitations. The study population was chosen from a specific healthcare provider, so it is a relatively specific population that cannot be assumed to be representative, as the socio-demographic characteristics of people with private insurance differ from those with statutory insurance [76]. For example, the study population has a relatively high level of education, so it can be assumed that there is a better understanding of the need for diverse changes. In addition, a “post-randomization consent design” according to Zelen [46] was chosen for the randomisation process, which is not entirely uncontroversial and has both advantages and disadvantages. The disadvantages of this design are typically different participation rates in the groups and differences in the baseline. In the present study, however, there were no serious differences in the baseline, but there were different participation rates, with a higher participation rate in the IG, despite the higher time and personal investment. This can be attributed to the potentially greater perceived benefit and at the same time shows an advantage of this design. This method was used to avoid the potential dissatisfaction of those who were not assigned to the intervention group which could have occurred in a randomisation after informed consent. However, a certain motivational bias cannot be completely ruled out, especially since there is also no information available as to whether there are characteristic differences between those who have consented to the study and those who have not. Also, an attrition bias cannot be completely ruled out, as the overall lost-to-follow-up rate is 23%. Last, it should be noted that in the subgroup analysis, the median self-efficacy is very high, so statistical ceiling effects cannot be ruled out.

However, the study also has some significant strengths. First, it is a randomised controlled trial and meets a very good scientific standard. In addition, the long duration of 2 years and the associated follow-up after 1 year should be mentioned positively. Studies in this area often have a brief duration; thus, little is yet known about long-term effects. Finally, another strength is the explicit recording of self-management and self-efficacy, which is still done too rarely.

## Conclusion

The present study has demonstrated that theory-based lifestyle interventions are capable of producing long-term effects on glycemic control, self-management, and self-efficacy in adults with type 2 diabetes. Here, self-efficacy in particular plays a major role in terms of treatment success and the maintenance of interventions. Given the dramatically increasing prevalence of diabetes and recent evidence of the large role of self-efficacy as an enhancer of interventions, including non-pharmacological interventions, this type of lifestyle intervention should be more strongly encouraged [16, 20, 23, 77]. Ideally, there should be nationwide access to lifestyle interventions for all affected people with type 2 diabetes. As this study has shown, this does not have to be geographically bound, but can also work very well and sustainably over distance. The expansion and promotion of digital services and the proactive approach to those affected will therefore play a major role in the future. In addition, future studies should continue to focus more on the long-term effects and implementation in real-life conditions. A good theoretical framework helps to create comparability here.

## Data Availability

The data does not belong to the university, but to the private health insurance company. We had a contractually agreed right of use for the research, which also includes publication. Unfortunately, however, we cannot provide the raw data itself, which is why there is no statement on this in the text, nor should there be.
